# How Can Programs Better Support Female Sex Workers to Avoid HIV Infection in Zimbabwe? A Prevention Cascade Analysis

**DOI:** 10.1097/QAI.0000000000001980

**Published:** 2019-02-07

**Authors:** Elizabeth Fearon, Andrew Phillips, Sibongile Mtetwa, Sungai T. Chabata, Phillis Mushati, Valentina Cambiano, Joanna Busza, Sue Napierala, Bernadette Hensen, Stefan Baral, Sharon S. Weir, Brian Rice, Frances M. Cowan, James R. Hargreaves

**Affiliations:** a Department of Social and Environmental Health Research, Public Health and Policy, London School of Hygiene and Tropical Medicine, London, United Kingdom;; b Institute for Global Health, Faculty of Population Health Sciences, University College London, London, United Kingdom;; c Centre for Sexual Health and HIV AIDS Research (CeSHHAR) Zimbabwe, Harare, Zimbabwe;; d Women's Global Health Imperative, RTI International, San Francisco, CA;; e Department of Clinical Research, Faculty of Infectious and Tropical Diseases, London School of Hygiene and Tropical Medicine, London, United Kingdom;; f Bloomberg School of Public Health, Johns Hopkins University, Baltimore, MD;; g Department of Epidemiology, Gillings School of Global Public Health, University of North Carolina at Chapel Hill, Chapel Hill, NC; and; h Department of International Public Health, Liverpool School of Tropical Medicine, Liverpool, United Kingdom.

**Keywords:** HIV prevention, condoms, pre-exposure prophylaxis, sex workers, sub-Saharan Africa, Zimbabwe

## Abstract

**Background:**

“HIV prevention cascades” have been proposed to support programs by identifying gaps in demand for, access to, and capability to adhere to HIV prevention tools, but there are few empirical examples to guide development. We apply a prevention cascade framework to examine prevention coverage and factors associated with condoms and/or PrEP adherence among female sex workers.

**Setting:**

Seven sites across Zimbabwe.

**Methods:**

Seven respondent-driven sampling surveys from the intervention sites of a pragmatic cluster-randomized trial in Zimbabwe in 2016 were analyzed, and 611/1439 women testing HIV-negative included. We operationalized key components of an HIV prevention cascade including demand, supply, and capability to adhere to 2 tools for HIV prevention: condoms and pre-exposure prophylaxis (PrEP). We used adjusted logistic regression to identify determinants of adherence to condoms and PrEP in turn, examining the effect of adherence to one tool on adherence to the other.

**Results:**

There were 343/611, 54.7%, women reporting adherence to condoms and/or PrEP, leaving almost half uncovered. Although women were aware that condoms prevented HIV and reported good access to them, only 45·5% reported full adherence to condom use. For PrEP, a new technology, there were gaps along all 3 domains of demand, supply, and adherence. Alcohol use decreased adherence to PrEP and condoms. Younger and newer entrants to sex work were less likely to take PrEP every day.

**Conclusions:**

HIV prevention programming among female sex workers in Zimbabwe could consider increasing awareness of PrEP alongside supply, alcohol use interventions, and approaches to engaging younger women.

## Abstract 2

Supplemental Digital Content is Available in the Text.

## INTRODUCTION

UNAIDS has set ambitious goals for reducing global HIV incidence through its HIV Prevention 2020 framework.^1^ Meeting these targets requires increasing coverage of populations at risk of HIV acquisition, including female sex workers (FSWs). Programs will need to ensure that demand for primary HIV prevention is high, evidence-based and rights-affirming HIV prevention tools are available and accessible, and adherence to prevention tools over time is monitored and supported.

In Zimbabwe, sex work is illegal and stigmatized, and FSWs are at high risk of HIV. Incidence has been estimated at 10% per year^2^ and site HIV prevalence estimates range between 40% and 80%.^3,4^ Regionally, HIV prevalence is 13.5 times higher among FSWs than among all women aged 15–49 years.^5^ Structural factors including poverty and economic shocks, criminalization, and stigma interact to raise the risk of HIV acquisition among FSWs through causal pathways affecting their vulnerability to violence, ability to negotiate with clients, access, carry and use condoms, and receive services and sensitive health care.^6–10^

Two tools that HIV-negative FSWs could use to reduce their risk of acquiring HIV are (1) consistently taking pre-exposure prophylaxis (PrEP) and (2) using condoms consistently. To increase the proportion of FSWs effectively using these prevention tools, programs will need to achieve 3 aims. First, they must ensure that there is high “demand” for these tools among FSWs by supporting them to perceive their risk of HIV, providing information and education about their effectiveness, and working toward a normative environment that supports their use. Second, programs will need to ensure that FSWs have geographic, financial, and stigma-free access to these tools (“supply”). Third, programs will need to work to ensure FSWs are capable of using the tools consistently over the period when they are at risk of acquiring HIV, which may require addressing both individual and structural factors that could inhibit adherence. These 3 “steps”—demand, supply and capability to adhere—have been proposed as an “HIV prevention cascade” analogous to the HIV treatment cascade, to help programs identify gaps in HIV prevention programs, to target their efforts, and to select among possible interventions.

Although templates for HIV prevention cascades have been suggested for individual prevention tools,^11–15^ programs need to understand how use of different prevention tools might interact with each other, for instance, in terms of risk compensation or with respect to how experience with one tool might affect demand for another, and how individual FSW characteristics might influence coverage. In this study, we apply a novel “dual” prevention cascade framework to measure the extent to which HIV-negative FSWs from 7 sites in Zimbabwe had demand for, were supplied with, and reported adherence to 2 prevention tools: either condoms and/or PrEP. Previous studies have found that lower levels of condom use among FSWs are associated with alcohol consumption,^16^ unsupportive relationships with other FSWs,^17^ experience of violence, and police harassment.^18^ Condom use can differ by partner type (spouse or steady partner versus a commercial client) and strength of relationship with clients.^19,20^ Once introduced to PrEP conceptually, FSWs have expressed high interest in using it,^21^ although as yet, there is limited evidence on factors influencing PrEP adherence among FSWs specifically. Among men who have sex with men and transgender women, adherence to PrEP has varied by structural factors including race, education, and economic security.^22–24^ Across demonstration trials, being younger than 30 years was found in meta-analysis to be associated with lower PrEP adherence.^25^

Here, we examine where there are gaps in support for prevention, and which FSW characteristics and experiences are associated with adherence to condoms and to PrEP. We identify a number of limitations to our approach based on secondary data and discuss these in detail, hoping that we will inspire others to continue to strengthen the data available for prevention cascades. Nevertheless, based on our findings, we make recommendations for strengthening HIV prevention in Zimbabwe's national sex worker HIV program.

## METHODS

### Setting and Population

This study is a secondary analysis including HIV-negative FSWs from 7 sites, which formed the intervention arm of the Sisters Antiretroviral Programme for Prevention of HIV: an Integrated Response (SAPPH-IRe) trial. This was a cluster (site)-randomized trial of an enhanced HIV care and prevention package for FSWs in 14 sites reflecting different sex work location types, including towns, growth points, collieries, and army bases. In all sites, the national sex work “Sisters with a Voice” programme (Sisters) provided free condoms and contraception, HIV testing and counseling, syndromic management of STIs, health education, community mobilization, and legal advice. In the 7 intervention sites, community mobilization was enhanced, clinical services to initiate ART and PrEP were available onsite, and community-based support for ART and PrEP adherence was provided. PrEP was offered to all women testing HIV-negative from July 2014 (November 2014 in one site) until endline in May 2016, along with a peer-based support programme and active follow-up. At this time in Zimbabwe, the SAPPH-IRe trial was the only way FSWs could access PrEP.

Cross-sectional respondent-driven sampling (RDS) surveys of approximately 200 women per site were conducted at study endline, with sample size determined by the primary trial outcome.^26^ Women were eligible if they had sold sex for money in the past 30 days, were aged 18 or older, and had been living/working in the site for 6 months. Because SAPPH-IRe was a pragmatic trial, we used RDS to obtain population-representative estimates among FSWs at each site to assess the impact of the intervention on the FSW population as a whole, not only those who had had some contact with the enhanced Sisters intervention. We describe detailed procedures elsewhere.^27^ Following mapping at each site, we purposefully selected initial “seeds” of 6 or 8 women, issued 2 coupons for recruitment, and reached 5 sample waves. Interviewers administered the questionnaire and entered data onto tablet computers, uploaded to a master database daily. A capillary blood sample was collected on dried blood spot for HIV antibody testing and, if reactive, HIV viral load measured.

### Measures

HIV status was assessed using the AniLabsytems EIA kit (AniLabsystems Ltd., OyToilette 3, FIN-01720, Finland) and confirmed by detectable viral load using NucliSENS EasyQ HIV-1 v2.0, or a second confirmatory ELISA (Enzygnost Anti-HIV 1/2 Plus ELISA; Dade Behring, Marburg, Germany) if no viral load was detected, but the antibody test was positive.

Participants self-reported sociodemographic characteristics, FSW social network size, and sex work characteristics. For the prevention cascade analysis, we defined measures of adherent condom use or adherent PrEP use, denoting “coverage” by reporting the use of one or both prevention tools. We asked women to recall condom use with steady partners and clients over different periods (last sex and previous month), and used prompting questions for women reporting “always” using them to confirm this. For the primary analyses, we denoted women as “adherent to condoms” if they reported no instance of condomless sex: at last vaginal sex, last anal sex, last sex with a client, nor in describing frequency of condom use with clients in the past month, at last sex with a steady partner not reported to be known as HIV-negative, and not in describing frequency of condom use in the last month with a steady partner not known as HIV-negative. For PrEP, we considered FSWs as adherent if they self-reported that they were currently taking PrEP, and that they were taking it every day.

Next, we identified variables related to the concepts of “demand” and supply'. In relation to demand for PrEP, we used self-reported data on whether women had heard of PrEP (recognizing this is only one dimension of demand). For condoms, we identified women who reported that condoms can prevent them from getting HIV, again recognizing that knowledge is a component of demand^28^ available in our data but does not describe it entirely. In relation to PrEP supply, we identified women who reported ever having been offered PrEP in the RDS survey. In relation to condoms supply, we measured whether women reported that condoms were “easily available” to them whenever needed. We recognize and discuss a number of limitations with these variables in Discussion and make recommendations for improvements in future efforts.

We identified variables that may be associated with demand, supply, and adherence to condoms and/or PrEP. We examined sociodemographic and sex work characteristics; frequency of alcohol consumption and binge drinking (6 or more alcoholic drinks in one night) in the previous 12 months; whether FSWs reported “good” or “very good” relations with other FSWs (concepts investigated in previous studies^29^), whether they discussed health with other FSWs and were encouraged by them; recent experience of being stopped by the police (further Zimbabwe context^30^); violence; and stigma related to being a sex worker (investigated in a previous study^31^). In assessing condom adherence, we also considered source of condoms (Sisters clinic, peer educator, clients) whether women were stopped by the police for carrying condoms, had refused a client who was drunk or violent, or had not used a condom because they were drunk, or because a client was drunk.

### Analytic Approaches

We have reported RDS diagnostics elsewhere.^27^ For these analyses, we further assessed whether site-specific estimates of condom and PrEP adherence appeared to converge over the recruitment waves (see Appendix 1, Supplemental Digital Content 1, http://links.lww.com/QAI/B282).

We described the sociodemographic and sex work characteristics of women testing HIV-negative at time of interview. In describing the prevention cascade, we pooled data from across the 7 sites but also reported the range of site specific estimates. We used RDS-II weighting when calculating proportions and in regression analyses, dropping seed participants, and weighting each woman in each site by the inverse of her “degree,” which we normalized by site when pooling data. We developed a “dual” HIV prevention cascade, including both condoms and PrEP. We estimated the proportion of HIV-negative women who “demanded,” were “supplied,” and who were able to “adhere” to condoms and/or PrEP, and therefore the proportion of all HIV-negative women who were “covered” by either or both HIV prevention method.

To guide the Sisters programme in improving HIV prevention coverage, we examined associations between FSW characteristics and experiences and their reported adherence to condoms and to PrEP. We included factors found in previous research among FSWs to determine condom use or those hypothesized to affect adherence to PrEP and included adherence to PrEP in the model for adherence to condoms and vice versa. We used logistic regression, dropping seed participants, weighting by site-normalized inverse degree and including a fixed term for site. We present crude associations and associations adjusted for age, education, marital status, food insecurity, age started sex work, and number of clients in the previous week.

We examined whether associations differed for adherence to condoms with clients or with steady partners, among those reporting steady partners. We also conducted our analyses without weighting for normalized inverse degree (see Appendix 2, Supplemental Digital Content 1, http://links.lww.com/QAI/B282).

All analyses were conducted using R version 3.3.2.

### Ethics

The SAPPH-IRe trial, including these analyses, received approval from the Medical Research Council Zimbabwe, University College London, the London School of Hygiene and Tropical Medicine, and RTI International.

### Role of the Funding Source

The funder of the study had no role in study design, data collection, data analysis, data interpretation, or writing of the report.

## RESULTS

### Recruitment

There were 611 HIV-negative FSWs among 1439 women recruited to the 7 intervention sites in 2016. RDS recruitment worked well, and convergence of adherence and HIV measures was achieved in most sites (see Appendix 1, Supplemental Digital Content 1, http://links.lww.com/QAI/B282 and trial report).^27^

### Description of Participants and Experience of Sex Work

Mean age among the women was 30.4 years. The majority of women had completed no or primary education only (68.2%) and were divorced/separated (63.1%), Table 1. Most women began sex work after age 20 (67.6%) and had 1-5 clients per week (60.3%). The majority reported “good” or “very good” relations with other FSWs (71.8%) and almost all agreed or strongly agreed that they felt comfortable discussing health issues with other FSWs (96.8%). Similar proportions reported that they experienced physical violence from intimate partners or clients in the past 1 month: 13.3% and 12.8%, respectively. There were 63.4% who reported that “they had been talked badly about” for being a sex worker and 29.2% said they had felt “ashamed” of being a sex worker. Three-percent reported being denied health services because they were sex workers. Almost half reported no alcohol consumption in the previous year; however, 16.5% reported drinking 4 or more nights per week and 25.7% reported drinking more than 6 drinks in one night at least once in the past 12 months. There were 9.7% and 10.3%, respectively, who reported that their own or client drinking had prevented them from using a condom at least once in the previous year.

**TABLE 1.**
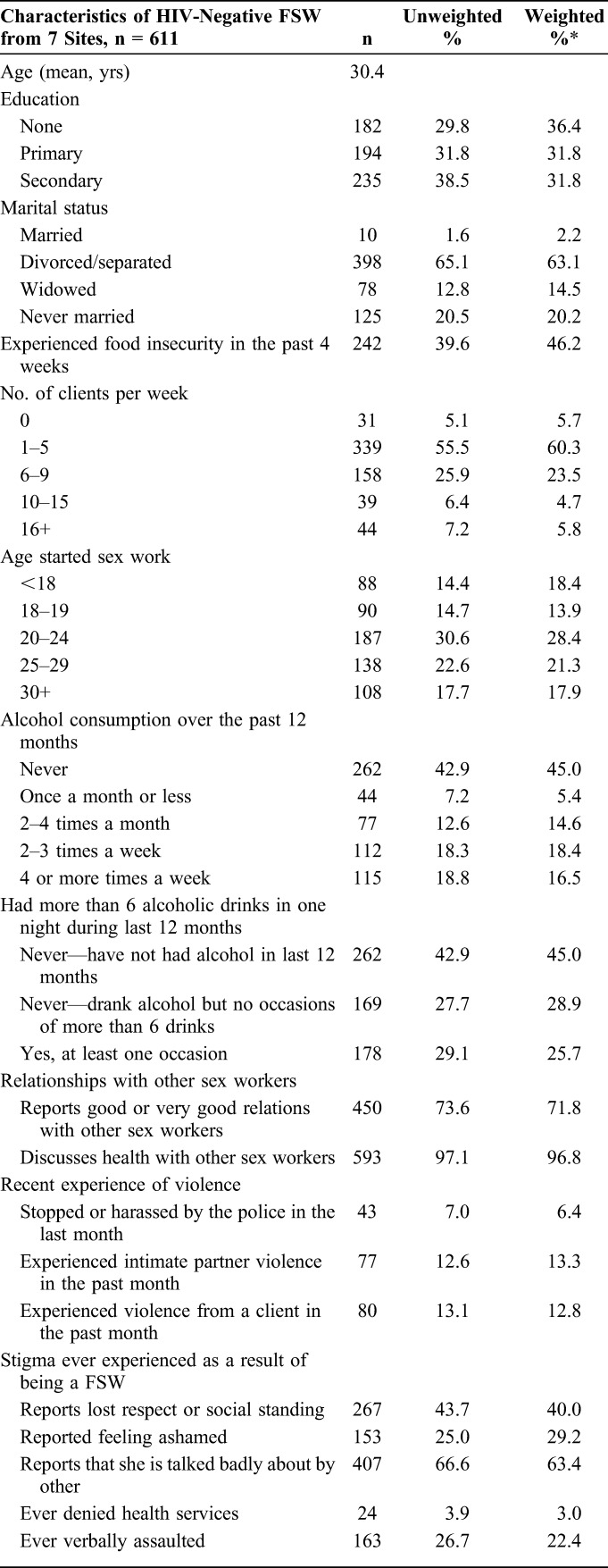

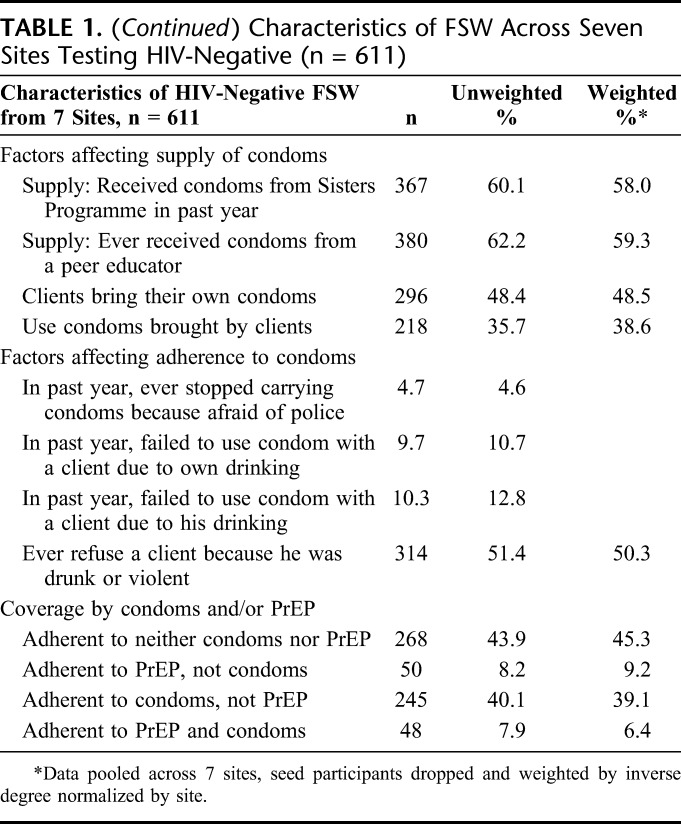
Characteristics of FSW Across Seven Sites Testing HIV-Negative (n = 611)

### Coverage of HIV Prevention: Demand, Supply, and Adherence to Condoms and/or PrEP

**FIGURE 1.**
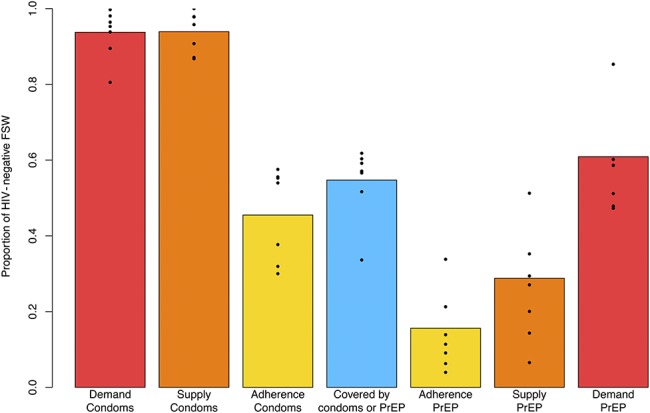
Demand, supply, adherence, and coverage by condoms and/or PrEP among 611 HIV-negative FSW from 7 sites. Data from 7 sites is pooled, weighted by inverse degree normalized by site with seed participants dropped. Points indicate site-specific estimates.

An estimated 54.7% of HIV-negative FSWs (site range 33.6%–61.8%) were either adherent to condom condoms and/or PrEP, Table 1 and Figure 1. Most (39.1% of all HIV-negative women) were using condoms consistently, but not taking and adherent to PrEP. There were 9.2% who were taking PrEP every day but not adherent to condoms, whereas 6.4% were adherent to both condoms and PrEP.

Some 94.0% of women reported that they knew that condoms could prevent HIV infection, and that they could access condoms, Figure 1. The proportion of women reporting that they were always adherent to condoms across all condom use questions was 45.5% (site range 30.0%–57.5%).

Some 60.9% of HIV-negative women had ever heard of PrEP, whereas 28.8% of HIV-negative women had ever been offered it. There were 15.6% of all HIV-negative women who reported currently taking PrEP and taking it every day.

### Measures of Condom Adherence

**FIGURE 2.**
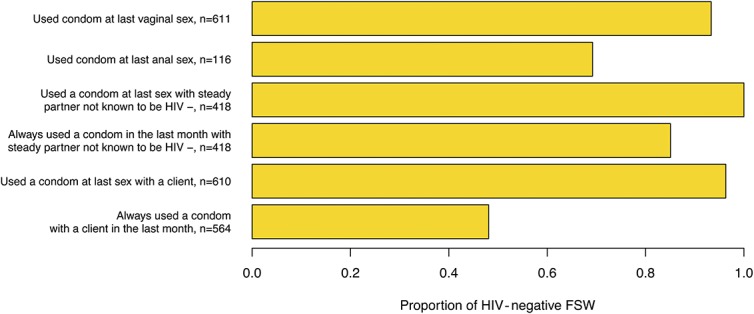
A comparison of measures of condom adherence among 611 HIV-negative FSW from 7 sites. Bar values indicate estimates from 7 sites that are pooled, weighted by inverse degree normalized by site with seed participants dropped. Not all measures applied to all women, (eg, Depending on whether the woman reported having a steady partner or declined to answer the question). The summary condom adherence measure is based on no reporting of noncondom use for any of the above measures. There were no participants for whom all variables were missing and the only measure with significant missingness was “always used a condom with a client in the last month,” which 47 participants declined to answer.

Levels of condom adherence varied depending on the measure chosen, Figure 2. Use at “last sex” measures were higher than measures asking about use over the previous month, which had an additional prompt for those initially answering that they had “always” used a condom. Although 96.3% of women said they had used a condom at last sex with a client, only 50.4% said that they had “always” used condoms with clients over the last month, confirmed by a prompt question. Adherence with steady partners not known to be HIV-negative was 85.1%, of the 418 women who reported steady partners. Across partner types and ways of asking about condom use, the weighted percentage of women who reported no instance of condomless sex, except with a steady partner known to be HIV-negative, was 45.5%.

### Factors Associated With Condom Adherence

Before adjustment, each additional year of age was associated with higher odds of condom adherence [crude odds ratio (OR) = 1.04, 95% confidence interval (CI): 1.02 to 1.07], as was starting sex work at an older age, Table 2. After adjustment, some evidence remained that starting sex work at an older age increased the likelihood of condom adherence [adjusted odds ratio (aOR) = 1.05, 95% CI: 1.00 to 1.11]. We did not find strong evidence for an association between condom adherence and education, marital status, food insecurity, relationships with other sex workers, or experience of stigma. Unadjusted, there was an association between being stopped or harassed by the police in the past month and reporting nonadherence to condoms (OR = 0.40, 95% CI: 0.17 to 0.94), but the evidence for this association reduced once adjusted (aOR = 0.50, 95% CI: 0.21 to 1.20). FSWs who had experienced client violence in the past month were also less likely to report condom adherence (crude OR = 0.46, 95% CI: 0.23 to 0.92), but after adjustment, the evidence for this association also reduced (aOR = 0.51, 95% CI: 0.25 to 1.23).

**TABLE 2.**
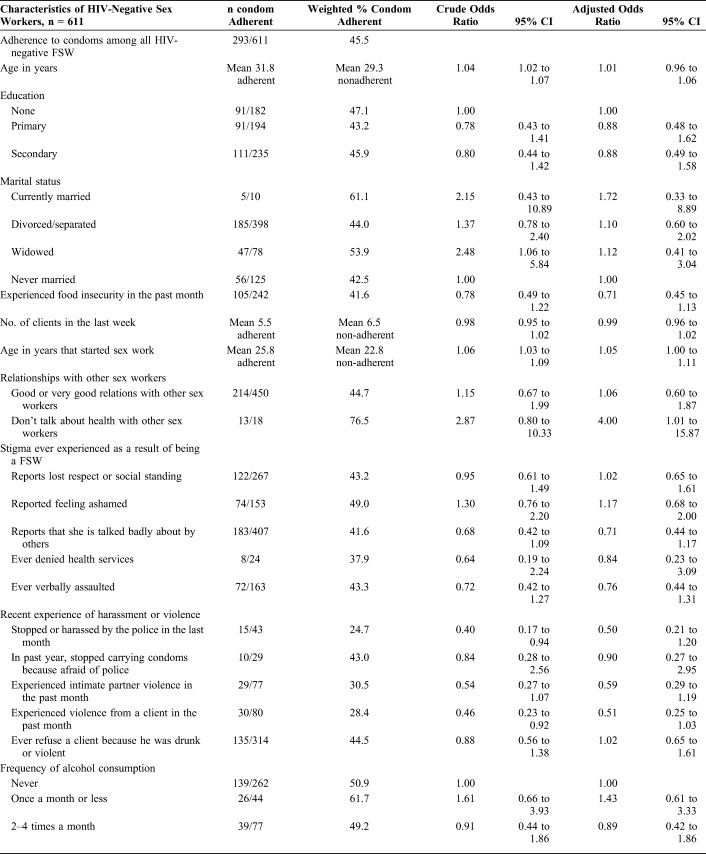

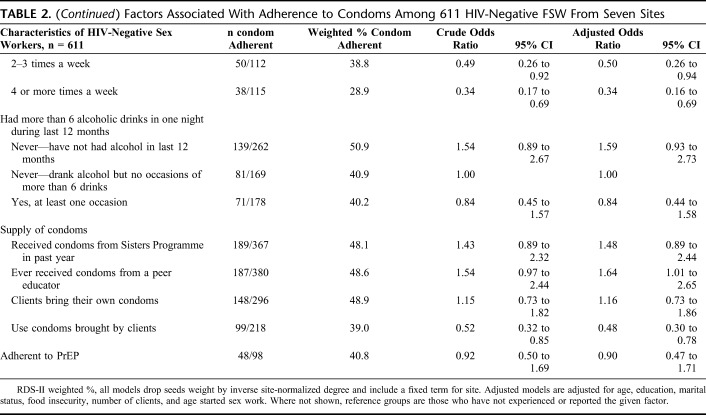
Factors Associated With Adherence to Condoms Among 611 HIV-Negative FSW From Seven Sites

Frequency of alcohol consumption, although not binge drinking, was associated with decreased condom adherence. After adjustment, women who reported that a client's drinking had prevented condom use had 0.22 times the odds of adherence compared with those who did not report this (95% CI: 0.07 to 0.64). Women who reported drinking alcohol 2 to 3 times or 4 or more times per week were also less likely to be adherent, (aOR = 0.34, 95% CI: 0.16 to 0.69 for 4 or more times, compared with no drinking).

Women who had received condoms from a peer educator were more likely to be adherent than those who had not (aOR = 1.64, 95% CI: 1.01 to 2.65). Women who reported using condoms brought by clients were less likely to adhere to them than those who did not, (aOR = 0.48, 95% CI: 0.30 to 0.78).

### Factors Associated With Adherence to PrEP

Women reporting adherent use of PrEP were more likely to be older, aOR = 1.05 for each additional year of age (95% CI: 1.01 to 1.10), but to have begun sex work at a younger age, aOR = 0.94 (95% CI: 0.89 to 0.99) for each year, indicating they had a longer duration of sex work than those nonadherent to PrEP, Table 3.

**TABLE 3.**
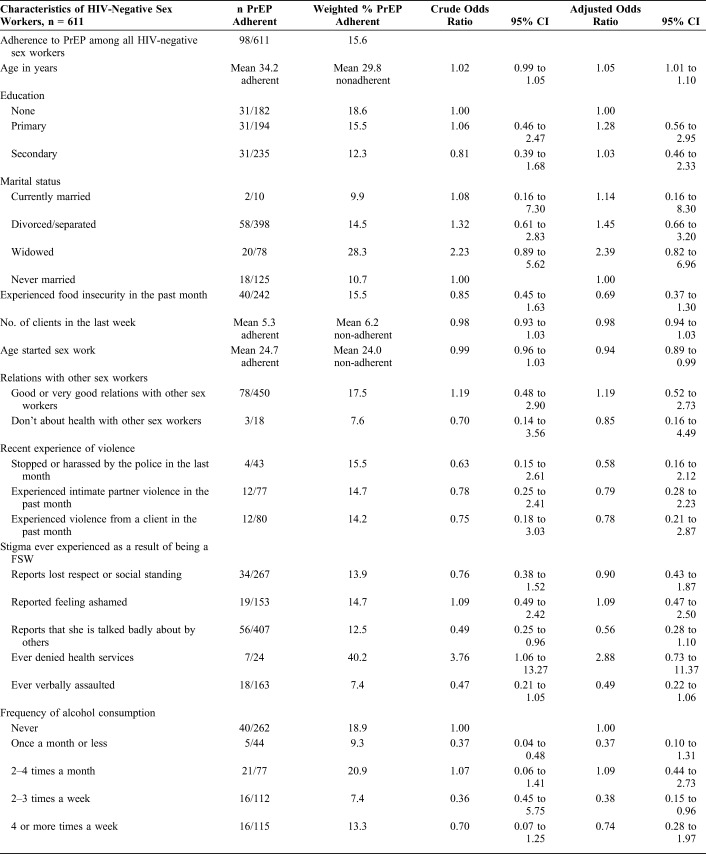

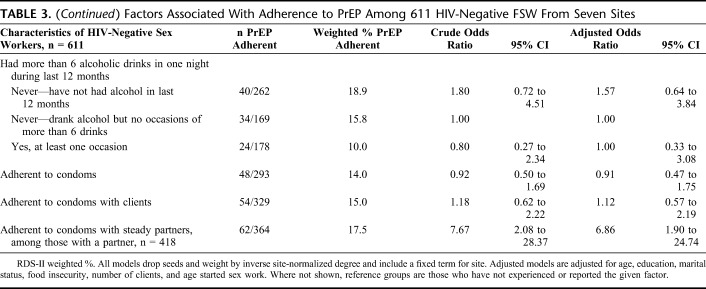
Factors Associated With Adherence to PrEP Among 611 HIV-Negative FSW From Seven Sites

Women who had alcohol 2–3 times per week were less likely to adhere to PrEP than those who never drank (aOR = 0.38, 95% CI: 0.15 to 0.96), although there was not a clear dose–response relationship, and there was no evidence for decreased adherence among those reporting binge drinking compared with those who drank alcohol but who did not report binge drinking. Adherence to condoms with all partners or with clients only was not associated with PrEP adherence. However, among those women who had steady partners, reported adherence to condoms with those partners was associated with increased likelihood of also being adherent to PrEP (aOR = 6.86, 95% CI: 1.90–24.74), Appendix 2, Supplemental Digital Content 1, Table 4 http://links.lww.com/QAI/B282.

### Sensitivity Analyses

There were 47 women missing responses to frequency of condom use with clients in the past month, which seemed to be differential by PrEP adherence. We repeated our analyses (1) without weighting participant respondents by normalized inverse degree; (2) with a different treatment for a missing condom use variable; and (3) examining condom adherence with clients and steady partners separately. These results are reported in full in Appendix 2, Supplemental Digital Content 1, http://links.lww.com/QAI/B282, but did not alter the overall conclusions from the primary analysis.

## DISCUSSION

We used an HIV prevention cascade framework^11^ to investigate levels of prevention coverage among HIV-negative FSWs at 7 sites in Zimbabwe in 2016. Approximately half of HIV-negative FSWs were currently adherent to condoms and/or to PrEP; almost half of HIV-negative FSWs are in need of additional strategies to prevent them from acquiring HIV.

Nearly all FSWs were aware that condoms could prevent HIV, an aspect of demand, and were able to access condoms when needed, an aspect of supply. However, there were gaps in adherence: condom use at all occasions, except with a steady partner believed to be HIV-negative, was reported by less than half of women (45.5%). A minority of women reported high alcohol consumption, but this was associated with nonadherence to condoms, and some sex workers reported that own or client alcohol use had caused them to have sex without a condom in the past year. Among Kenyan FSW, an adaptation of WHO's Brief Intervention for Hazardous and Harmful Drinking reduced alcohol use^32^ and experience of client violence, which could be applicable for FSW in Zimbabwe.^33^ Programming could consider how to support women to use condoms even in situations where they and/or their clients are drinking. Although our study found weak statistical evidence for an association between condom adherence and experience of violence and police harassment, alcohol consumption and experiencing violence and harassment have been found to be related in other FSW populations^34^ and should be explored further.

Women whose clients provided condoms were less likely to be adherent than those who did not, whereas women who received condoms from a peer educator were more likely to be adherent. FSW depending more on clients could have had a less reliable and trustworthy supply in practice. Women who meet peer educators are given condom negotiation training and education, which could additionally benefit their condom adherence.

Our measurement of condom adherence confirms the recommendation to use multiple questions in measuring coverage of condom use.^35^ The UNAIDS Global AIDS Monitoring indicator of condom use among FSW—condom use at last sex with a client^3^—measured adherence at 96%, whereas this dropped to 48% when asking whether women had always used condoms in the previous month. Our findings point to the need for caution when applying this indicator to constructing prevention cascades for FSW, which could give a false impression of high condom adherence.

For PrEP, as expected for a new tool (and in this case available only as part of a trial), there were gaps across demand, supply, and adherence. Programmes might need to support younger and newer entrants to sex work to take up and adhere to PrEP, as well as those women with a higher alcohol consumption, the latter also a concern identified by FSW in Kenya.^36^ PrEP is more likely than condoms to be taken at a time other than when alcohol is being consumed, which might be an advantage. However, our data use a cross-sectional measure of adherence, and while other studies of FSW have found strong interest in PrEP once FSW are made aware of it, they highlight the need for long-term support to take it.^37,38^

Our findings point to the importance of considering prevention tools together in a dual prevention cascade. It is important to understand whether women who are not able to use condoms consistently are able to use PrEP. There are also fears of “risk compensation” in relation to PrEP usage, whereby those on PrEP increase their frequency of condomless sex, although the evidence for changes in sexual risk behaviors, reported condom use, and STIs among men who have sex with men, and transgender women starting PrEP has been mixed.^23,39–41^ Overall, we did not find a statistically significant relationship between condom and PrEP adherence except among women with steady partners in our study, where condom adherence with partners not known to be HIV-negative was associated with a higher likelihood of PrEP adherence than condom nonadherence. These women might have been more capable of adhering to prevention in general. However, there was possible differential condom use reporting bias by PrEP adherence status, making conclusions about how PrEP and condom use interact difficult. We need longitudinal cohort studies and ongoing monitoring to better determine how women use condoms and PrEP, why they choose one or the other, and whether this varies by partner type and other circumstances.

This is a secondary analysis, and there are limitations with the application of a prevention cascades framework to these data. A core aim of our work was to try to operationalize the prevention cascade framework, and to reflect on limitations and suggest improvements for future applications. Concepts of demand and supply are multidimensional and are not fully described by the variables available here. We used having heard of PrEP and awareness of condoms as preventing HIV infection as necessary, but not sufficient, measures of demand. Other factors hypothesized to affect demand, such as encouragement to take PrEP by other sex workers, are included in our risk factor analyses, but we did not measure individual risk perception or make more detailed assessment of norms. We considered supply measures from the perspective of individual sex workers rather than examining programme outputs, for example. In future applications, it could be beneficial to consider programme and user perspectives in tandem^13^ to assess whether they align. We did find some variation in cascade components across sites, particularly for PrEP. Our data are from intervention sites of a cluster-randomized trial and might not be generalizable to a later roll-out of PrEP in this population, although the trial was pragmatic and thus closer to routine delivery than an efficacy trial.

As strengths, our data were collected from a diverse group of sites using identical protocols and RDS, designed to be representative of the population of sex workers, unlike data from small, non–population-based demonstration projects. Although our outcomes were self-reported and subject to reporting biases, we were able to biologically determine which women were HIV-negative.

In future applications of the prevention cascade, more nuanced data describing concepts of demand (knowledge, attitudes, perceived risk, and normative environment) and supply could be developed. Measuring demand in the context of multipurpose products such as condoms should also be considered. It might not be the case that these concepts are best measured using a single quantitative survey, and methods such as discrete choice experiments^42^ and participatory ranking^43^ might be informative, as well as combining data from programme records and surveys. Future applications might also consider these intermediate cascade steps as outcomes to understand what factors are particularly associated with demand for or supply of HIV-prevention tools. Zimbabwe has a PrEP implementation plan for which roll-out has begun,^44^ and as PrEP usage expands, analyses of the differences between subgroups of those covered by no prevention tools, covered by both PrEP and condoms, or covered by either PrEP or condoms could help to further understand which subgroups might adopt which prevention strategy and in what circumstances.

We have shown a dual cascade HIV prevention framework of demand, supply, and adherence to be informative in determining levels of prevention coverage among FSW at high risk of HIV acquisition, and in identifying programmatic gaps and possible strategies. In line with a combination prevention approach, we recommend that prevention cascades consider demand, supply, and capability to adhere to different prevention tools together and investigate the role of structural, community, and individual-level factors in determining coverage.

## Supplementary Material

SUPPLEMENTARY MATERIAL

## References

[1] Dehne KL Dallabetta G Wilson D HIV Prevention 2020: a framework for delivery and a call for action. Lancet HIV. 2016;3:e323–e332. 27365207 10.1016/S2352-3018(16)30035-2

[2] Hargreaves JR Mtetwa S Davey C Implementation and operational research: cohort analysis of program data to estimate HIV incidence and uptake of HIV-related services among female sex workers in Zimbabwe, 2009–2014. J Acquir Immune Defic Syndr. 2016;72:e1–e8. 27093516 10.1097/QAI.0000000000000920

[3] Cowan FM Davey C Fearon E The HIV care cascade among female sex workers in Zimbabwe: results of a population-based survey from the Sisters Antiretroviral therapy Programme for Prevention of HIV, an Integrated Response (SAPPH-IRe) Trial. J Acquir Immune Defic Syndr. 2017;74:375–382. 27930599 10.1097/QAI.0000000000001255

[4] Cowan FM Mtetwa S Davey C Engagement with HIV prevention treatment and care among female sex workers in Zimbabwe: a respondent driven sampling survey. PLoS One. 2013;8:e77080. 24143203 10.1371/journal.pone.0077080 PMC3797143

[5] Baral S Beyrer C Muessig K Burden of HIV among female sex workers in low-income and middle-income countries: a systematic review and meta-analysis. Lancet Infect Dis. 2012;12:538–549. 22424777 10.1016/S1473-3099(12)70066-X

[6] Shannon K Strathdee SA Goldenberg SM Global epidemiology of HIV among female sex workers: influence of structural determinants. Lancet. 2015;385:55–71. 25059947 10.1016/S0140-6736(14)60931-4 PMC4297548

[7] Shannon K Strathdee SA Shoveller J Structural and environmental barriers to condom use negotiation with clients among female sex workers: implications for HIV-prevention strategies and policy. Am J Public Health. 2009;99:659–665. 19197086 10.2105/AJPH.2007.129858 PMC2661482

[8] Scorgie F Nakato D Harper E “We are despised in the hospitals”: sex workers' experiences of accessing health care in four African countries. Cult Health Sex. 2013;15:450–465. 23414116 10.1080/13691058.2012.763187

[9] Decker MR Crago AL Chu SK Human rights violations against sex workers: burden and effect on HIV. Lancet. 2015;385:186–199. 25059943 10.1016/S0140-6736(14)60800-X PMC4454473

[10] Beattie TS Bhattacharjee P Isac S Declines in violence and police arrest among female sex workers in Karnataka state, south India, following a comprehensive HIV prevention programme. J Int AIDS Soc. 2015;18:20079. 26477992 10.7448/IAS.18.1.20079 PMC4609649

[11] Hargreaves JR Delany-Moretlwe S Hallett TB The HIV prevention cascade: integrating theories of epidemiological, behavioural, and social science into programme design and monitoring. Lancet HIV. 2016;3:e318–e322. 27365206 10.1016/S2352-3018(16)30063-7

[12] Horn T Sherwood J Remien RH Towards an integrated primary and secondary HIV prevention continuum for the United States: a cyclical process model. J Int AIDS Soc. 2016;19:21263. 27863535 10.7448/IAS.19.1.21263 PMC5116064

[13] Garnett GP Hallett TB Takaruza A Providing a conceptual framework for HIV prevention cascades and assessing feasibility of empirical measurement with data from east Zimbabwe: a case study. Lancet HIV. 2016;3:e297–e306. 27365204 10.1016/S2352-3018(16)30039-X PMC4935672

[14] Nunn AS Brinkley-Rubinstein L Oldenburg CE Defining the HIV pre-exposure prophylaxis care continuum. AIDS. 2017;31:731–734. 28060019 10.1097/QAD.0000000000001385 PMC5333727

[15] Weiner R Fineberg M Dube B Using a cascade approach to assess condom uptake in female sex workers in India: a review of the Avahan data. BMC Public Health. 2018;18:897. 30029597 10.1186/s12889-018-5842-6 PMC6053780

[16] Chersich MF Bosire W King'ola N Effects of hazardous and harmful alcohol use on HIV incidence and sexual behaviour: a cohort study of Kenyan female sex workers. Glob Health. 2014;10:22. 10.1186/1744-8603-10-22 PMC3985581 24708844

[17] Qiao S Li X Zhang C Social support and condom use among female sex workers in China. Health Care Women Int. 2015;36:834–850. 25365752 10.1080/07399332.2014.971952 PMC6234009

[18] Erausquin JT Reed E Blankenship KM Change over time in police interactions and HIV risk behavior among female sex workers in Andhra Pradesh, India. AIDS Behav. 2015;19:1108–1115. 25354735 10.1007/s10461-014-0926-5 PMC4512652

[19] Kayembe PK Mapatano MA Busangu AF Determinants of consistent condom use among female commercial sex workers in the Democratic Republic of Congo: implications for interventions. Sex Transm Infect. 2008;84:202–206. 18055581 10.1136/sti.2007.028324

[20] Deering KN Bhattacharjee P Bradley J Condom use within non-commercial partnerships of female sex workers in southern India. BMC Public Health. 2011;11(suppl 6):S11. 10.1186/1471-2458-11-S6-S11 PMC3287549 22376171

[21] Koechlin FM Fonner VA Dalglish SL Values and preferences on the use of oral pre-exposure prophylaxis (PrEP) for HIV prevention among multiple populations: a systematic review of the literature. AIDS Behav. 2017;21:1325–1335. 27900502 10.1007/s10461-016-1627-z PMC5378753

[22] Marcus JL Hurley LB Hare CB Preexposure prophylaxis for HIV prevention in a large integrated health care system: adherence, renal safety, and discontinuation. J Acquir Immune Defic Syndr. 2016;73:540–546. 27851714 10.1097/QAI.0000000000001129 PMC5424697

[23] Liu AY Cohen SE Vittinghoff E Preexposure prophylaxis for HIV infection integrated with municipal- and community-based sexual health services. JAMA Intern Med. 2016;176:75–84. 26571482 10.1001/jamainternmed.2015.4683 PMC5042323

[24] Hoagland B Moreira RI De Boni RB High pre-exposure prophylaxis uptake and early adherence among men who have sex with men and transgender women at risk for HIV infection: the PrEP Brasil demonstration project. J Int AIDS Soc. 2017;20:21472. 28418232 10.7448/IAS.20.1.21472 PMC5515021

[25] Yun K Xu JJ Zhang J Female and younger subjects have lower adherence in PrEP trials: a meta-analysis with implications for the uptake of PrEP service to prevent HIV. Sex Transm Infect. 2017;94:163–168. 28756409 10.1136/sextrans-2017-053217

[26] Hargreaves JR Fearon E Davey C Statistical design and analysis plan for an impact evaluation of an HIV treatment and prevention intervention for female sex workers in Zimbabwe: a study protocol for a cluster randomised controlled trial. Trials. 2016;17:6. 26728882 10.1186/s13063-015-1095-1 PMC4700631

[27] Cowan F Davey C Fearon E Targeted combination prevention to support female sex workers in Zimbabwe accessing and adhering to antiretrovirals for treatment and prevention of HIV (SAPPH-IRe): a cluster-randomised trial. Lancet HIV 2018;5:e417–e26. 10.1016/S2352-3018(18)30111-5 30030134

[28] Kahle EM Sullivan S Stephenson R Functional knowledge of pre-exposure prophylaxis for HIV prevention among participants in a web-based survey of sexually active gay, Bisexual, and other men who have sex with men: cross-sectional study. JMIR Public Health Surveill. 2018;4:e13. 29362213 10.2196/publichealth.8089 PMC5801519

[29] Mtetwa S Busza J Davey C Competition is not necessarily a barrier to community mobilisation among sex workers: an intervention planning assessment from Zimbabwe. BMC Public Health. 2015;15:787. 26275906 10.1186/s12889-015-2118-2 PMC4537541

[30] Busza J Mtetwa S Fearon E Good news for sex workers in Zimbabwe: how a court order improved safety in the absence of decriminalization. J Int AIDS Soc. 2017;20:21860. 28530034 10.7448/IAS.20.1.21860 PMC5515058

[31] Hargreaves JR Busza J Mushati P Overlapping HIV and sex-work stigma among female sex workers recruited to 14 respondent-driven sampling surveys across Zimbabwe, 2013. AIDS Care. 2017;29:675–685. 27998178 10.1080/09540121.2016.1268673

[32] L'Engle KL Mwarogo P Kingola N A randomized controlled trial of a brief intervention to reduce alcohol use among female sex workers in Mombasa, Kenya. J Acquir Immune Defic Syndr. 2014;67:446–453. 25197826 10.1097/QAI.0000000000000335

[33] Parcesepe AM KL LE Martin SL The impact of an alcohol harm reduction intervention on interpersonal violence and engagement in sex work among female sex workers in Mombasa, Kenya: results from a randomized controlled trial. Drug Alcohol Depend. 2016;161:21–28. 26872880 10.1016/j.drugalcdep.2015.12.037 PMC4936780

[34] Deering KN Amin A Shoveller J A systematic review of the correlates of violence against sex workers. Am J Public Health. 2014;104:e42–e54. 10.2105/AJPH.2014.301909 PMC3987574 24625169

[35] Fonner VA Kennedy CE O'Reilly KR Systematic assessment of condom use measurement in evaluation of HIV prevention interventions: need for standardization of measures. AIDS Behav. 2014;18:2374–2386. 24197972 10.1007/s10461-013-0655-1 PMC4013255

[36] Mack N Evens EM Tolley EE The importance of choice in the rollout of ARV-based prevention to user groups in Kenya and South Africa: a qualitative study. J Int AIDS Soc. 2014;17(3 suppl 2):19157. 25224616 10.7448/IAS.17.3.19157 PMC4164014

[37] Eakle R Gomez GB Naicker N HIV pre-exposure prophylaxis and early antiretroviral treatment among female sex workers in South Africa: results from a prospective observational demonstration project. PLoS Med. 2017;14:e1002444. 29161256 10.1371/journal.pmed.1002444 PMC5697804

[38] Mboup A Béhanzin L Guédou FA Early antiretroviral therapy and daily pre-exposure prophylaxis for HIV prevention among female sex workers in Cotonou, Benin: a prospective observational demonstration study. J Int AIDS Soc. 2018;21:e25208. 10.1002/jia2.25208 PMC6287093 31291057

[39] Hoornenborg E Krakower DS Prins M Pre-exposure prophylaxis for MSM and transgender persons in early adopting countries. AIDS. 2017;31:2179–2191. 28991023 10.1097/QAD.0000000000001627 PMC5812254

[40] Lal L Audsley J Murphy DA Medication adherence, condom use and sexually transmitted infections in Australian preexposure prophylaxis users. AIDS. 2017;31:1709–1714. 28700394 10.1097/QAD.0000000000001519

[41] Grant RM Anderson PL McMahan V Uptake of pre-exposure prophylaxis, sexual practices, and HIV incidence in men and transgender women who have sex with men: a cohort study. Lancet Infect Dis. 2014;14:820–829. 25065857 10.1016/S1473-3099(14)70847-3 PMC6107918

[42] Quaife M Eakle R Cabrera Escobar MA Divergent preferences for HIV prevention: a discrete choice experiment for multipurpose HIV prevention products in South Africa. Med Decis Making. 2018;38:120–133. 28863752 10.1177/0272989X17729376

[43] Mavhu W Langhaug L Pascoe S A novel tool to assess community norms and attitudes to multiple and concurrent sexual partnering in rural Zimbabwe: participatory attitudinal ranking. AIDS Care. 2011;23:52–59. 21218276 10.1080/09540121.2010.490257

[44] Implementation Plan for HIV Pre-exposure Prophylaxis in Zimbabwe 2018–2020. Harare, Zimbabwe: Ministry of Health and Child Care; 2018.

