# Supplementary material for: How Can Programs Better Support Female Sex Workers to Avoid HIV Infection in Zimbabwe? A Prevention Cascade Analysis
Source: J Acquir Immune Defic Syndr. 2019 Feb 7;81(1):24–35. doi: 10.1097/QAI.0000000000001980 (PMC6467580; doi:10.1097/QAI.0000000000001980)
Supplement: SUPPLEMENTARY MATERIAL [file qai-81-024-s001.docx]

# Appendix 1: Respondent Driven Sampling Diagnostics

We have reported in detail on the RDS diagnostics for these surveys in relation to the main SAPPH-IRe trial finding^1^. Here, we considered recruitment dynamics as they related to the prevention cascade framework, examining recruitment trees by adherence to condoms and to PrEP, and the extent to which estimates of adherence to condoms and PrEP had converged as the sample accumulated. As recruitment included both HIV-positive and negative FSW, we include all in our diagnostics report (n=1439).

PrEP and condom adherence did not appear to concentrate in any particular recruitment chains (example tree, Figure 1). RDS-weighted estimates for adherence to PrEP appeared to converge well in each site (Figure 3), and adherence to condoms well in four sites (Figure 2). In three sites, it is possible the adherence to condoms estimate might have risen further with additional recruitment, and therefore we might have underestimated condom adherence when pooling the data.

**Appendix 1 Figure 1 : One example site’s recruitment trees by HIV status and A) adherence to condoms; and B) adherence to PrEP among all FSW at the seven sites (n=1439)**

*Circles are participants and lines are recruitment ties. The top of each chain is the seed participant, with her recruits below and so on.*

**A B**

**Appendix 1 Figure 2: Convergence of estimates of adherence to condoms in seven sites**

*For each plot, the solid line shows the cumulative RDS-II weighted estimate of adherence to condoms as the sample size increased. The dotted line shows the final estimate.*

*Note that estimates are out of all women in these denominators (n=1439 total recruited), not only those 611 HIV-negative, as in the main analysis.*

**** ****

**** ****

**Appendix 1 Figure 3: Convergence of estimates of adherence to PrEP in seven sites**

*For each plot, the solid line shows the cumulative RDS-II weighted estimate of adherence to condoms as the sample size increased. The dotted line shows the final estimate. Note that estimates are out of all women in these denominators (n=1439 total recruited), not only those n=611 HIV-negative, as in the main analysis.*

**** ****

# Appendix 2: Sensitivity Analysis Results

We examined how consistent our findings as to factors associated with condom and PrEP adherence were when 1) observations were unweighted (seed participants still dropped); 2) 47 women who declined to report condom use with clients over the last month were excluded from the analyses rather than coded according to their other condom use responses; and 3) adherence to condoms with clients and adherence to condoms with steady partners were treated separately.

#### Unweighted analyses

The unweighted analyses showed less evidence that using condoms provided by peer educators was associated with higher adherence (aOR=1.12, 95% CI 0.78-1.61 unweighted, compared to aOR=1.64 (95% CI 1.01-2.65) weighted. There was also not evidence that women who did not talk about health with other sex workers were more likely to be adherent to condoms, an association seen in the weighted model. Women who do not talk about health with other sex workers were rare (18/611) and had a lower network size than women who did. This would have weighted their responses upwards, which could account for the difference with the weighted model. Other associations with alcohol use, duration in sex work and condom supply were similar across the weighted and unweighted models.

The unweighted model for PrEP adherence was consistent with the weighted model in identifying a possible association with alcohol use, but showed reduced evidence for an association between prep adherence, current age, age began sex work or condom adherence with a partner, Table 3 main paper and Table 2 Appendix 2.

#### Condom adherence with steady partners not known to be HIV-negative compared to condom adherence with clients

Women with steady partners (n=418) reported higher condom adherence with partners who were not known to be HIV-negative, than with clients (85.1% compared to 50.4%), Figure 2 main paper.

Alcohol use was associated with reduced condom adherence both with partners and with clients, Tables 5 and 6 Appendix 2. Women who had experienced violence from a client in the last month were less likely to report condom adherence with partners (aOR=0.25, 95% CI 0.09-0.69), though there was less evidence for this association with clients (aOR=0.53, 95% CI 0.26-1.06). Using condoms brought by clients was associated with client condom adherence (aOR 0.48, 95% CI 0.29-0.78), but not condom use among partners (aOR=0.86, 95% CI 0.36-2.06). Women who reported that they were talked badly about on account of being a sex worker were also less likely to be adherent to condoms with partners, but there was little evidence for this association with condom adherence with clients.

There was evidence that women who were adherent to PrEP were more likely to adhere to condoms with a partner, 97% among those adhering to PrEP, (aOR=7.97, 95% 1.76-36.08), but there was not evidence for this association with clients (aOR 1.14, 95% CI 0.58-2.23), Table 5 and 6 Appendix 2. This finding was reflected in the corresponding analysis using PrEP adherence as the outcome and condom adherence with partners as the exposure in the main paper, Table 3.

#### Excluding n=47 women who do not report frequency of condom use with clients in the last month

Women who were adherent to PrEP were more likely to have a missing response to this variable. In the primary study analysis, their condom adherence measure was coded according to their responses to the other condom use questions. However, because the question about frequency of use with clients in the past month was the most discriminatory, we repeated our risk factor analyses for condom adherence and PrEP adherence excluding these women. In so doing, there was a strong was effect of adhering to PrEP on adhering to condoms (aOR=0.57, 95% CI 0.29-1.13), Appendix 2 Table 5, than in the main analysis (aOR=0.90, 95% CI 0.47-1.71), Table 3 main paper, but the statistical evidence remained weak.

**Appendix 2 Table 1: Unweighted findings of factors associated with adherence to condoms among 611 HIV-negative FSW**

**Appendix 2 Table 2: Unweighted findings of factors associated with adherence to PrEP among 611 HIV-negative FSW**

**Appendix 2 Table 3: Associations with adherence to condoms, excluding women who do not report frequency of condom use with clients in the past month, n=564**

**Appendix 2 Table 4: Associations with adherence to PrEP, excluding women who do not report frequency of condom use with clients in the past month, n=564**

##

**Appendix Table 5: Adherence to condoms with clients amongst HIV-negative FSW, n=611**

**Appendix Table 6: Adherence to condoms with partners amongst HIV-negative FSW who have a steady partner, n=418**

**References**

1. Cowan F, Davey C, Fearon E, et al. Randomised trial of a combination intervention to empower female sex workers in Zimbabwe to link and adhere to antiretrovirals for treatment and prevention. *(Submitted).* 2017.
